# Cost savings of EMS administration of bronchodilators and systemic corticosteroids for pediatric asthma patients

**DOI:** 10.3389/fpubh.2025.1601926

**Published:** 2025-08-18

**Authors:** Jennifer Fishe, Gerard Garvan, Lauren Riney, Erik Finlay, Sam Palmer, Phyllis Hendry

**Affiliations:** ^1^Department of Emergency Medicine, College of Medicine, University of Florida, Jacksonville, FL, United States; ^2^Center for Data Solutions, College of Medicine, University of Florida, Jacksonville, FL, United States; ^3^Cincinnati Children’s Hospital Medical Center, College of Medicine, University of Cincinnati, Cincinnati, OH, United States; ^4^GeoPlan Center, University of Florida, Gainesville, FL, United States

**Keywords:** asthma, emergency medical services, pediatric asthma, pediatric emergency medicine, systemic corticosteroids

## Abstract

**Background:**

Pediatric asthma costs the United States healthcare system $5 billion annually. A major component of those costs are hospitalizations for acute exacerbations. This brief report examines the cost savings from emergency medical services (EMS) administration of bronchodilators and systemic corticosteroids to pediatric asthma patients, as opposed to waiting for emergency department (ED) arrival.

**Methods:**

This is an economic analysis of data from a study of seven EMS agencies who incorporated both bronchodilators and systemic corticosteroids in their standard operating protocols for all children ages 0–17 years experiencing an asthma exacerbation. Comparing hospital admission rates between children who did and did not receive those medications from EMS, we calculated the number needed to treat (NNT) to prevent one hospitalization, and using the most recently available cost estimate of a pediatric asthma hospitalization, we calculated cost savings for EMS administration of bronchodilators and systemic corticosteroids.

**Results:**

For all pediatric asthma patients, the NNT was 58.8, which would avert 1,913 hospitalizations nationwide and save $6,886,800. For mild severity exacerbations, the NNT was 9, and for patients with an EMS transport time longer than 40 min, the NNT was 6. If EMS administered bronchodilators and systemic corticosteroids to all mild severity exacerbation and prolonged transport time pediatric asthma patients, then 6,833 mild severity and 500 prolonged transport time patients could avert a hospitalization, resulting in savings of $26,398,800.

**Conclusion:**

EMS administration of bronchodilators and systemic corticosteroids results in cost savings, most notably for mild severity patients and those with prolonged transport times.

## 1 Introduction

This brief report details the economic implications of emergency medical services (EMS) administration of evidence-based treatments for pediatric asthma exacerbations. Asthma affects an estimated 4.5 million children in the United States (US) and results in over $5 billion in costs to the healthcare system (1, 2). A major component of those total healthcare costs are hospitalizations for acute exacerbations. Annual pediatric asthma hospitalization costs were estimated to be a total of $1.59 billion in 2009, and $3,600 per child hospitalized in 2010 (2).

Our research has focused on improving the prehospital EMS care of children with asthma exacerbations. EMS treats an estimated 1 million children every year in the US (3), and approximately 150,000 of those encounters are for asthma exacerbations (4). Therefore, EMS treats a significant number of pediatric asthma exacerbations, and there is an opportunity for EMS to provide clinical care that both improves patient outcomes and results in healthcare cost savings. This study addresses whether EMS administration of bronchodilators and systemic corticosteroids to children with asthma could result in healthcare cost savings.

## 2 Methods

Recently, our study team published a seven-site EMS agency study examining whether EMS administration of both bronchodilators and oral systemic corticosteroid to children with asthma exacerbations can decrease hospital admission rates (as opposed to waiting for arrival to the ED to receive those medications) (5). Briefly, that study was an observational, stepped-wedge design study examining outcomes overall and at each site before and after the EMS agencies incorporated oral systemic corticosteroids into their standard operating protocols for pediatric asthma. That study and this economic sub-analysis were approved by the study institution’s Institutional Review Board. Here we present an economic analysis of healthcare cost differences between patients who first received the bundle bronchodilators and systemic corticosteroids from EMS and those who received bronchodilators and systemic corticosteroids after ED arrival.

To perform this analysis, we calculated the difference in hospital admission rates between groups, which represented the absolute risk reduction between the EMS administration group and ED administration group. Using the absolute risk reduction, we calculated the number needed to treat (NNT) using the standard formula of:


$$\mathrm{NNT}=\frac{1}{\text{Absolute Risk Reduction}}$$

After calculating the NNT, we used the estimated number pediatric asthma patients treated by EMS (e.g., 150,000 annually) to calculate the number of hospitalizations that could be averted by EMS administration of the bundle of bronchodilators and systemic corticosteroids using the following formula:


$$\text{Hospitalizations averted}=\frac{\text{Number of pediatric asthma patients treated}}{\mathrm{NNT}}$$

To calculate healthcare cost savings, we used the most recent available estimates in the published literature from 2010, which estimated the average cost of a pediatric asthma hospitalization at $3,600.

Cost savings was therefore calculated as:


Cost savings=Hospitalizations avertedx$3,600

We performed those calculations for patients overall, and for mild severity exacerbation patients and those with transport times longer than 40 min, as those two sub-groups saw significant decreases in hospital admission rates. Of note, asthma severity in the seven-site study was determined using an EMS-specific score comprised of objective data, determined retrospectively from the EMS encounter records.

## 3 Results

We found that administration of both of bronchodilators and systemic corticosteroids by EMS, as opposed to waiting for ED arrival, decreased hospital admission rates from 32.6 to 30.9%, although the decrease was not statistically significant (5). That decrease represents an absolute risk reduction of 1.7% and a number needed to treat (NNT) of 58.8 (1/0.017 = 58.8) to prevent one hospitalization. The resulting cost savings for that seven-site study was $9,122.

However, in that same study, only 25% of patients received both treatments from EMS. Given that EMS treats an estimated 150,000 pediatric asthma patients annually, if the national EMS bronchodilator and systemic corticosteroid administration rate was 50% instead of 25% (i.e., 37,500 patients instead of 75,000 patients), 637 hospitalizations could be averted. If the rate was 100%, 1,913 hospitalizations could be averted. Using the most recent available comprehensive national data of the cost of a pediatric asthma-related hospitalization from 2010 (2), averting 1,913 hospitalizations would translate to $6,886,800 in healthcare cost savings.

In a sub-analysis by asthma exacerbation severity and EMS transport time, we found greater benefits of EMS administration of bronchodilators and systemic corticosteroids to certain subgroups, specifically mild severity exacerbations and patients with prolonged transport times (6). For mild severity exacerbation patients, EMS administration of bronchodilators and systemic corticosteroids resulted in a 10.9% decrease in hospitalizations, which is an absolute risk reduction of 10.9% and an NNT of 9. For patients with EMS to ED transport times longer than 40 min (“prolonged transport”), EMS administration of systemic corticosteroids and bronchodilators resulted in a 16.7% decrease in hospitalizations, for an absolute risk reduction of 16.7% and an NNT of 6.

Therefore, there could be a greater economic impact from targeted EMS administration of the bundle of bronchodilators and systemic corticosteroids to specific prehospital patients. Although the national proportion of mild exacerbation and prolonged transport time patients is unknown, in our seven-site study of 809 patients, 41% were of mild severity and 2% had transport times longer than 40 min. If EMS administered bronchodilators and systemic corticosteroids to all mild severity exacerbation and prolonged transport time pediatric asthma patients, then 6,833 mild severity and 500 prolonged transport time patients could avert a hospitalization, resulting in total savings of $26,398,800 (using data from 2010).

## 4 Discussion

The potential healthcare cost savings in 2024 dollars are likely much higher given inflation. Unfortunately, there are no more current published literature from which to draw pediatric asthma hospital cost estimates than the cited 2010 study. While millions of dollars are a small portion of total estimated $4.9 trillion in healthcare costs in the United States (7), there are additional, non-monetary benefits to averting hospitalizations for pediatric patients and their families, such as minimizing disruptions to school attendance, less missed days of work for caregivers, and reducing the risk of hospital acquired infections.

The findings regarding mild severity exacerbations are notable given that many EMS agencies’ standard operating protocols tailor medication recommendations based off severity. For example, a protocol may advise only bronchodilator administration for mild severity exacerbations, with systemic corticosteroids reserved for more severe exacerbations. Our economic results show that mild severity exacerbation patients can benefit from EMS administration of systemic corticosteroids, and should prompt re-evaluation of standard operating protocols that base medication recommendations off of severity. Perhaps it is the milder exacerbation patients for which early EMS administration of systemic corticosteroids has the greatest potential to avert a hospitalization (in contrast to a very severe exacerbation, where prompt EMS care may avert an intensive care unit admission but not a hospital admission). Additionally, our prior study found that 41% of exacerbations were of a mild severity (5), therefore a significant proportion of EMS pediatric asthma patients stand to benefit from prehospital administration of the bundle of bronchodilators and systemic corticosteroids.

Our prolonged transport time findings are of particular interest to rural EMS agencies. Patients with prolonged transport times likely reside in rural areas. Rural emergency healthcare is currently strained by the closures of critical access hospitals and consolidation of pediatric inpatient care, including for asthma (8), to larger urban centers. Therefore, rural EMS systems have a unique opportunity to provide timely, evidence-based care in the form of bronchodilators and systemic corticosteroids that can improve patient outcomes, while at the same time potentially improve rural ED operations by lessening the need for a subsequent interfacility transfer for pediatric inpatient admission.

These results are notable given the relatively low cost and ease of administration of bronchodilators and systemic corticosteroids, two medications which many EMS agencies stock for both adult and pediatric care. Additionally, given the now-frequent occurrence of medication shortages (9), there are multiple options for both bronchodilators and systemic corticosteroids for pediatric asthma exacerbation treatment, making inclusion of these medications in EMS agencies’ formulary flexible and feasible.

These results highlight the potential cost-savings and patient-centered benefits of early EMS administration of emergency medications for pediatric asthma exacerbations. While there is a growing body of EMS literature highlighting cost-savings of mobile integrated health and community paramedicine programs (10, 11), there is a paucity of studies on cost-savings for pediatric emergency treatments and more economic analyses of prehospital pediatric care are warranted.

This sub-analysis has limitations that merit consideration. Most notably, the most recent healthcare cost estimates available for the cost of pediatric asthma hospitalization are from 2010, and more recent data is needed to provide precise estimates. Additionally, hospitalization costs vary from region to region and facility to facility. This analysis does not include the cost of EMS stocking medications or EMS clinician training to administer them to children using weight-based dosing. This analysis includes seven EMS agencies, and therefore the findings may not be generalizable to all EMS agencies nationwide.

In conclusion, we found evidence that shifting first administration of bronchodilators and systemic corticosteroids from the ED to the prehospital setting for pediatric asthma patients treated by EMS resulted in cost savings and a reduction of hospitalizations, particularly for mild exacerbation patients and those with prolonged transport times. Depending on local EMS agency resources and the characteristics of their patient population, incorporation of both bronchodilators and systemic corticosteroids into EMS standard operating protocols is worthy of consideration.

## Data Availability

The data analyzed in this study is subject to the following licenses/restrictions: Data availability is subject to the terms of data use agreements with the participating EMS agencies. Requests to access these datasets should be directed to jennifer.fishe@jax.ufl.edu.
